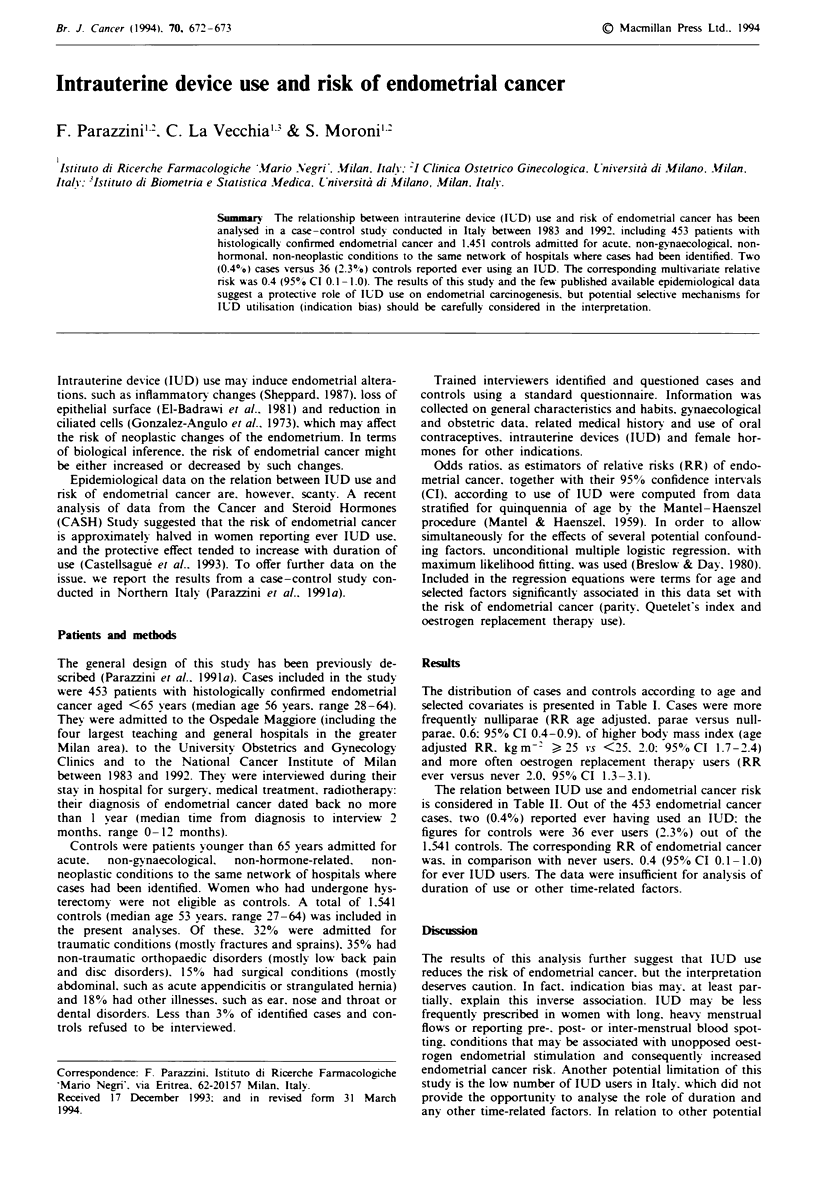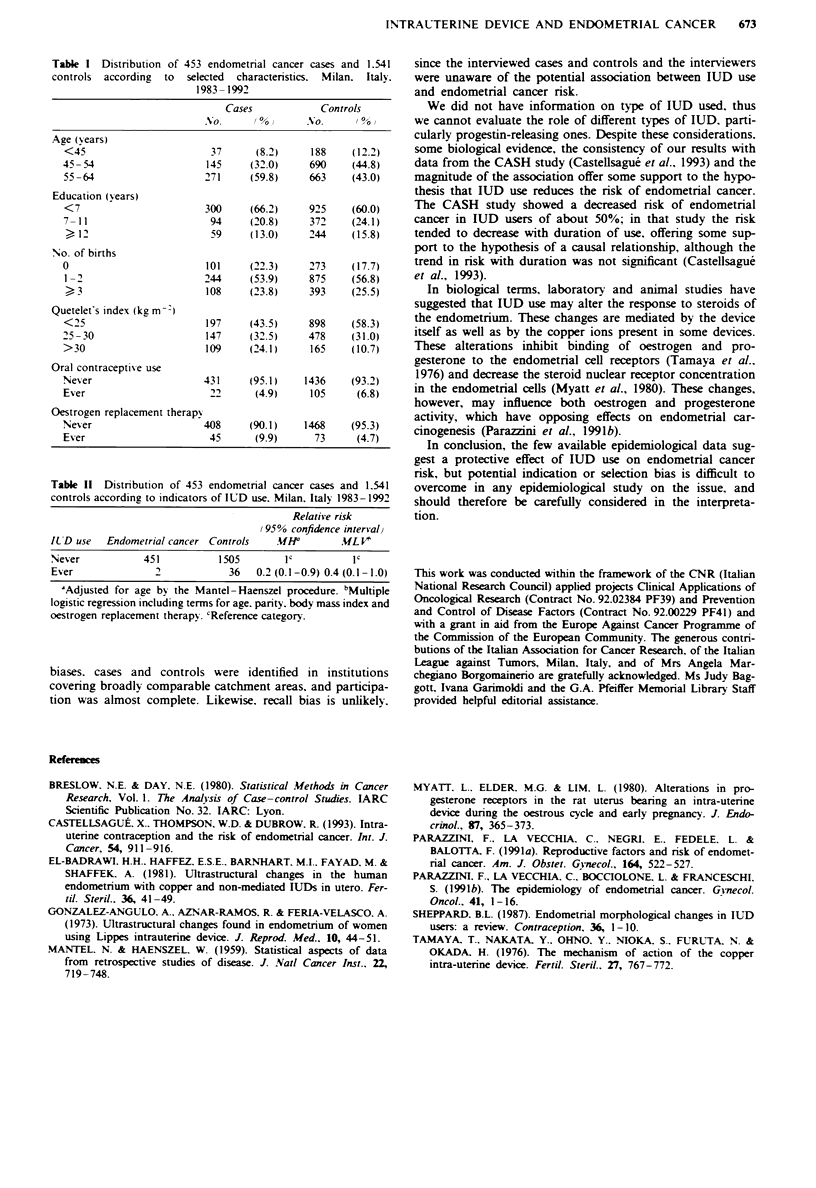# Intrauterine device use and risk of endometrial cancer.

**DOI:** 10.1038/bjc.1994.369

**Published:** 1994-10

**Authors:** F. Parazzini, C. La Vecchia, S. Moroni

**Affiliations:** Istituto di Ricerche Farmacologiche Mario Negri, Milan, Italy.

## Abstract

The relationship between intrauterine device (IUD) use and risk of endometrial cancer has been analysed in a case-control study conducted in Italy between 1983 and 1992, including 453 patients with histologically confirmed endometrial cancer and 1,451 controls admitted for acute, non-gynaecological, non-hormonal, non-neoplastic conditions to the same network of hospitals where cases had been identified. Two (0.4%) cases versus 36 (2.3%) controls reported ever using an IUD. The corresponding multivariate relative risk was 0.4 (95% CI 0.1-1.0). The results of this study and the few published available epidemiological data suggest a protective role of IUD use on endometrial carcinogenesis, but potential selective mechanisms for IUD utilisation (indication bias) should be carefully considered in the interpretation.


Br. J. Cancer (1994), 70, 672–673 © Macmillan Press Ltd., 1994

### Intrauterine device use and risk of endometrial cancer

F. Parazzini[1,2], C. La Vecchia[1,3] & S. Moroni[1,2]

[1]Istituto di Ricerche Farmacologiche 'Mario Negri', Milan, Italy; [2]I Clinica Ostetrico Ginecologica, Università di Milano, Milan, Italy; [3]Istituto di Biometria e Statistica Medica, Università di Milano, Milan, Italy.

**Summary** The relationship between intrauterine device (IUD) use and risk of endometrial cancer has been analysed in a case–control study conducted in Italy between 1983 and 1992, including 453 patients with histologically confirmed endometrial cancer and 1,451 controls admitted for acute, non-gynaecological, non-hormonal, non-neoplastic conditions to the same network of hospitals where cases had been identified. Two (0.4%) cases versus 36 (2.3%) controls reported ever using an IUD. The corresponding multivariate relative risk was 0.4 (95% CI 0.1–1.0). The results of this study and the few published available epidemiological data suggest a protective role of IUD use on endometrial carcinogenesis, but potential selective mechanisms for IUD utilisation (indication bias) should be carefully considered in the interpretation.

Intrauterine device (IUD) use may induce endometrial alterations, such as inflammatory changes (Sheppard, 1987), loss of epithelial surface (El-Badrawi *et al.*, 1981) and reduction in ciliated cells (Gonzalez-Angulo *et al.*, 1973), which may affect the risk of neoplastic changes of the endometrium. In terms of biological inference, the risk of endometrial cancer might be either increased or decreased by such changes.

Epidemiological data on the relation between IUD use and risk of endometrial cancer are, however, scanty. A recent analysis of data from the Cancer and Steroid Hormones (CASH) Study suggested that the risk of endometrial cancer is approximately halved in women reporting ever IUD use, and the protective effect tended to increase with duration of use (Castellsagué *et al.*, 1993). To offer further data on the issue, we report the results from a case–control study conducted in Northern Italy (Parazzini *et al.*, 1991*a*).

### Patients and methods

The general design of this study has been previously described (Parazzini *et al.*, 1991*a*). Cases included in the study were 453 patients with histologically confirmed endometrial cancer aged <65 years (median age 56 years, range 28–64). They were admitted to the Ospedale Maggiore (including the four largest teaching and general hospitals in the greater Milan area), to the University Obstetrics and Gynecology Clinics and to the National Cancer Institute of Milan between 1983 and 1992. They were interviewed during their stay in hospital for surgery, medical treatment, radiotherapy: their diagnosis of endometrial cancer dated back no more than 1 year (median time from diagnosis to interview 2 months, range 0–12 months).

Controls were patients younger than 65 years admitted for acute, non-gynaecological, non-hormone-related, non-neoplastic conditions to the same network of hospitals where cases had been identified. Women who had undergone hysterectomy were not eligible as controls. A total of 1,541 controls (median age 53 years, range 27–64) was included in the present analyses. Of these, 32% were admitted for traumatic conditions (mostly fractures and sprains), 35% had non-traumatic orthopaedic disorders (mostly low back pain and disc disorders), 15% had surgical conditions (mostly abdominal, such as acute appendicitis or strangulated hernia) and 18% had other illnesses, such as ear, nose and throat or dental disorders. Less than 3% of identified cases and controls refused to be interviewed.

Trained interviewers identified and questioned cases and controls using a standard questionnaire. Information was collected on general characteristics and habits, gynaecological and obstetric data, related medical history and use of oral contraceptives, intrauterine devices (IUD) and female hormones for other indications.

Odds ratios, as estimators of relative risks (RR) of endometrial cancer, together with their 95% confidence intervals (CI), according to use of IUD were computed from data stratified for quinquennia of age by the Mantel–Haenszel procedure (Mantel & Haenszel, 1959). In order to allow simultaneously for the effects of several potential confounding factors, unconditional multiple logistic regression, with maximum likelihood fitting, was used (Breslow & Day, 1980). Included in the regression equations were terms for age and selected factors significantly associated in this data set with the risk of endometrial cancer (parity, Quetelet's index and oestrogen replacement therapy use).

### Results

The distribution of cases and controls according to age and selected covariates is presented in Table I. Cases were more frequently nulliparae (RR age adjusted, parae versus nulliparae, 0.6; 95% CI 0.4–0.9), of higher body mass index (age adjusted RR, kg $m^{-2}$ $\geqslant 25$ *vs* $<25$, 2.0; 95% CI 1.7–2.4) and more often oestrogen replacement therapy users (RR ever versus never 2.0, 95% CI 1.3–3.1).

The relation between IUD use and endometrial cancer risk is considered in Table II. Out of the 453 endometrial cancer cases, two (0.4%) reported ever having used an IUD; the figures for controls were 36 ever users (2.3%) out of the 1,541 controls. The corresponding RR of endometrial cancer was, in comparison with never users, 0.4 (95% CI 0.1–1.0) for ever IUD users. The data were insufficient for analysis of duration of use or other time-related factors.

### Discussion

The results of this analysis further suggest that IUD use reduces the risk of endometrial cancer, but the interpretation deserves caution. In fact, indication bias may, at least partially, explain this inverse association. IUD may be less frequently prescribed in women with long, heavy menstrual flows or reporting pre-, post- or inter-menstrual blood spotting, conditions that may be associated with unopposed oestrogen endometrial stimulation and consequently increased endometrial cancer risk. Another potential limitation of this study is the low number of IUD users in Italy, which did not provide the opportunity to analyse the role of duration and any other time-related factors. In relation to other potential

Correspondence: F. Parazzini, Istituto di Ricerche Farmacologiche 'Mario Negri', via Eritrea, 62-20157 Milan, Italy.
Received 17 December 1993; and in revised form 31 March 1994.

**Table I** Distribution of 453 endometrial cancer cases and 1,541 controls according to selected characteristics, Milan, Italy, 1983–1992

| | Cases | | Controls | |
|---|---|---|---|---|
| | *No.* | *(%)* | *No.* | *(%)* |
| Age (years) | | | | |
| <45 | 37 | (8.2) | 188 | (12.2) |
| 45–54 | 145 | (32.0) | 690 | (44.8) |
| 55–64 | 271 | (59.8) | 663 | (43.0) |
| Education (years) | | | | |
| <7 | 300 | (66.2) | 925 | (60.0) |
| 7–11 | 94 | (20.8) | 372 | (24.1) |
| ⩾12 | 59 | (13.0) | 244 | (15.8) |
| No. of births | | | | |
| 0 | 101 | (22.3) | 273 | (17.7) |
| 1–2 | 244 | (53.9) | 875 | (56.8) |
| ⩾3 | 108 | (23.8) | 393 | (25.5) |
| Quetelet's index (kg m$^{-2}$) | | | | |
| <25 | 197 | (43.5) | 898 | (58.3) |
| 25–30 | 147 | (32.5) | 478 | (31.0) |
| >30 | 109 | (24.1) | 165 | (10.7) |
| Oral contraceptive use | | | | |
| Never | 431 | (95.1) | 1436 | (93.2) |
| Ever | 22 | (4.9) | 105 | (6.8) |
| Oestrogen replacement therapy | | | | |
| Never | 408 | (90.1) | 1468 | (95.3) |
| Ever | 45 | (9.9) | 73 | (4.7) |

**Table II** Distribution of 453 endometrial cancer cases and 1,541 controls according to indicators of IUD use, Milan, Italy 1983–1992

| | | | Relative risk (95% confidence interval) | |
|---|---|---|---|---|
| *IUD use* | *Endometrial cancer* | *Controls* | *MH*[a] | *MLV*[b] |
| Never | 451 | 1505 | 1[c] | 1[c] |
| Ever | 2 | 36 | 0.2 (0.1–0.9) | 0.4 (0.1–1.0) |

[a]Adjusted for age by the Mantel–Haenszel procedure. [b]Multiple logistic regression including terms for age, parity, body mass index and oestrogen replacement therapy. [c]Reference category.

biases, cases and controls were identified in institutions covering broadly comparable catchment areas, and participation was almost complete. Likewise, recall bias is unlikely, since the interviewed cases and controls and the interviewers were unaware of the potential association between IUD use and endometrial cancer risk.

We did not have information on type of IUD used, thus we cannot evaluate the role of different types of IUD, particularly progestin-releasing ones. Despite these considerations, some biological evidence, the consistency of our results with data from the CASH study (Castellsagué *et al.*, 1993) and the magnitude of the association offer some support to the hypothesis that IUD use reduces the risk of endometrial cancer. The CASH study showed a decreased risk of endometrial cancer in IUD users of about 50%; in that study the risk tended to decrease with duration of use, offering some support to the hypothesis of a causal relationship, although the trend in risk with duration was not significant (Castellsagué *et al.*, 1993).

In biological terms, laboratory and animal studies have suggested that IUD use may alter the response to steroids of the endometrium. These changes are mediated by the device itself as well as by the copper ions present in some devices. These alterations inhibit binding of oestrogen and progesterone to the endometrial cell receptors (Tamaya *et al.*, 1976) and decrease the steroid nuclear receptor concentration in the endometrial cells (Myatt *et al.*, 1980). These changes, however, may influence both oestrogen and progesterone activity, which have opposing effects on endometrial carcinogenesis (Parazzini *et al.*, 1991*b*).

In conclusion, the few available epidemiological data suggest a protective effect of IUD use on endometrial cancer risk, but potential indication or selection bias is difficult to overcome in any epidemiological study on the issue, and should therefore be carefully considered in the interpretation.

This work was conducted within the framework of the CNR (Italian National Research Council) applied projects Clinical Applications of Oncological Research (Contract No. 92.02384 PF39) and Prevention and Control of Disease Factors (Contract No. 92.00229 PF41) and with a grant in aid from the Europe Against Cancer Programme of the Commission of the European Community. The generous contributions of the Italian Association for Cancer Research, of the Italian League against Tumors, Milan, Italy, and of Mrs Angela Marchegiano Borgomainerio are gratefully acknowledged. Ms Judy Baggott, Ivana Garimoldi and the G.A. Pfeiffer Memorial Library Staff provided helpful editorial assistance.

### References

BRESLOW, N.E. & DAY, N.E. (1980). *Statistical Methods in Cancer Research*, Vol. 1. *The Analysis of Case–control Studies*. IARC Scientific Publication No. 32. IARC: Lyon.

CASTELLSAGUÉ, X., THOMPSON, W.D. & DUBROW, R. (1993). Intrauterine contraception and the risk of endometrial cancer. *Int. J. Cancer*, **54**, 911–916.

EL-BADRAWI, H.H., HAFFEZ, E.S.E., BARNHART, M.I., FAYAD, M. & SHAFFEK, A. (1981). Ultrastructural changes in the human endometrium with copper and non-mediated IUDs in utero. *Fertil. Steril.*, **36**, 41–49.

GONZALEZ-ANGULO, A., AZNAR-RAMOS, R. & FERIA-VELASCO, A. (1973). Ultrastructural changes found in endometrium of women using Lippes intrauterine device. *J. Reprod. Med.*, **10**, 44–51.

MANTEL, N. & HAENSZEL, W. (1959). Statistical aspects of data from retrospective studies of disease. *J. Natl Cancer Inst.*, **22**, 719–748.

MYATT, L., ELDER, M.G. & LIM, L. (1980). Alterations in progesterone receptors in the rat uterus bearing an intra-uterine device during the oestrous cycle and early pregnancy. *J. Endocrinol.*, **87**, 365–373.

PARAZZINI, F., LA VECCHIA, C., NEGRI, E., FEDELE, L. & BALOTTA, F. (1991*a*). Reproductive factors and risk of endometrial cancer. *Am. J. Obstet. Gynecol.*, **164**, 522–527.

PARAZZINI, F., LA VECCHIA, C., BOCCIOLONE, L. & FRANCESCHI, S. (1991*b*). The epidemiology of endometrial cancer. *Gynecol. Oncol.*, **41**, 1–16.

SHEPPARD, B.L. (1987). Endometrial morphological changes in IUD users: a review. *Contraception*, **36**, 1–10.

TAMAYA, T., NAKATA, Y., OHNO, Y., NIOKA, S., FURUTA, N. & OKADA, H. (1976). The mechanism of action of the copper intra-uterine device. *Fertil. Steril.*, **27**, 767–772.